# Normal variation of magnetic resonance T1 relaxation times in the human population at 1.5 T using ShMOLLI

**DOI:** 10.1186/1532-429X-15-13

**Published:** 2013-01-20

**Authors:** Stefan K Piechnik, Vanessa M Ferreira, Adam J Lewandowski, Ntobeko AB Ntusi, Rajarshi Banerjee, Cameron Holloway, Mark BM Hofman, Daniel M Sado, Viviana Maestrini, Steven K White, Merzaka Lazdam, Theodoros Karamitsos, James C Moon, Stefan Neubauer, Paul Leeson, Matthew D Robson

**Affiliations:** 1 Department of Cardiovascular Medicine, Oxford Centre for Clinical Magnetic Resonance Research, University of Oxford, Oxford, UK; 2 Stephenson Cardiovascular MR Centre, Libin Cardiovascular Institute of Alberta, Calgary, AL, Canada; 3 Department of Cardiovascular Medicine, Oxford Cardiovascular Clinical Research Facility, University of Oxford, Oxford, UK; 4 Department of Physics & Medical Technology, ICaR-VU, VU, University Medical Center, Amsterdam, the Netherlands; 5 The Heart Hospital Imaging Centre, The Heart Hospital, London and University College, London, UK

## Abstract

**Background:**

Quantitative T1-mapping is rapidly becoming a clinical tool in cardiovascular magnetic resonance (CMR) to objectively distinguish normal from diseased myocardium. The usefulness of any quantitative technique to identify disease lies in its ability to detect significant differences from an established range of normal values. We aimed to assess the variability of myocardial T1 relaxation times in the normal human population estimated with recently proposed Shortened Modified Look-Locker Inversion recovery (ShMOLLI) T1 mapping technique.

**Methods:**

A large cohort of healthy volunteers (n = 342, 50% females, age 11–69 years) from 3 clinical centres across two countries underwent CMR at 1.5T. Each examination provided a single average myocardial ShMOLLI T1 estimate using manually drawn myocardial contours on typically 3 short axis slices (average 3.4 ± 1.4), taking care not to include any blood pool in the myocardial contours. We established the normal reference range of myocardial and blood T1 values, and assessed the effect of potential confounding factors, including artefacts, partial volume, repeated measurements, age, gender, body size, hematocrit and heart rate.

**Results:**

Native myocardial ShMOLLI T1 was 962 ± 25 ms. We identify the partial volume as primary source of potential error in the analysis of respective T1 maps and use 1 pixel erosion to represent “midwall myocardial” T1, resulting in a 0.9% decrease to 953 ± 23 ms. Midwall myocardial ShMOLLI T1 was reproducible with an intra-individual, intra- and inter-scanner variability of ≤2%. The principle biological parameter influencing myocardial ShMOLLI T1 was the female gender, with female T1 longer by 24 ms up to the age of 45 years, after which there was no significant difference from males. After correction for age and gender dependencies, heart rate was the only other physiologic factor with a small effect on myocardial ShMOLLI T1 (6ms/10bpm). Left and right ventricular blood ShMOLLI T1 correlated strongly with each other and also with myocardial T1 with the slope of 0.1 that is justifiable by the resting partition of blood volume in myocardial tissue. Overall, the effect of all variables on myocardial ShMOLLI T1 was within 2% of relative changes from the average.

**Conclusion:**

Native T1-mapping using ShMOLLI generates reproducible and consistent results in normal individuals within 2% of relative changes from the average, well below the effects of most acute forms of myocardial disease. The main potential confounder is the partial volume effect arising from over-inclusion of neighbouring tissue at the manual stages of image analysis. In the study of cardiac conditions such as diffuse fibrosis or small focal changes, the use of “myocardial midwall” T1, age and gender matching, and compensation for heart rate differences may all help to improve the method sensitivity in detecting subtle changes. As the accuracy of current T1 measurement methods remains to be established, this study does not claim to report an accurate measure of T1, but that ShMOLLI is a stable and reproducible method for T1-mapping.

## Background

In magnetic resonance imaging, T1, also known as the spin–lattice relaxation time, is an intrinsic magnetic property of a tissue [1]. Each tissue type, including myocardium, exhibits a characteristic range of normal T1 relaxation times at a particular magnetic field strength [2], and deviation from the normal range may be indicative of disease. Cardiac T1-mapping without the use of exogenous contrast agents has been shown to be sensitive to a variety of pathologies, notably acute myocardial infarction [3,4], myocarditis [5] and cardiac amyloidosis [6], all of which demonstrate significant (i.e. 20-30%) increase in native T1 times. Although currently the study of diffuse fibrosis predominantly concentrates on post-contrast T1-mapping [7-9], native T1-mapping holds significant promise in this field [10,11].

In order to use native myocardial T1-mapping to accurately identify disease states, a stable normal range and factors that influence it need to be established. These include common physiologic variation in the normal population as well as technical factors inherent to the measurement technique and image analysis.

In this study, we aimed to establish the normal range, potential sources of error and confounds of native myocardial T1 relaxation times in a large cohort of healthy human volunteers. T1-mapping was performed at 1.5T using the Shortened Modified Look-Locker Inversion Recovery (ShMOLLI) technique [12]. We examined the effects of normal physiologic parameters, including age, gender, heart rate, weight, height and hematocrit, as well as technical factors, such as partial volume effects, myocardial thickness and inter-centre variability on identical scanners within and across three clinical centres in two countries.

## Methods

### Study population

Myocardial T1-mapping was performed in 342 subjects representative of a general healthy population (172 females:170 males, age 11–69 years) over 374 separate scans in three clinical MR centres: Oxford Centre for Clinical Magnetic Resonance, UK (OXF 297 scans), London Heart Hospital, UK (LON 64 scans) and VU University Medical Center, Amsterdam, The Netherlands (AMS 13 scans). All subjects were recruited through advertising as control cases for research studies. None had evidence of cardiovascular disease or cardiac risk factors including hypertension or diabetes, based on medical history. None were referred as patients for a clinical cardiovascular MR (CMR) scan which then turned out to be normal. In majority of cases the 12 lead ECG, blood pressure or selected blood tests were confirmed normal on the day of scan. There were no pathological findings identified in the available cine images. All study procedures were approved by the respective local ethics committees in each centre and all subjects gave written informed consent.

### MR acquisition parameters

All T1 measurements were performed without the administration of any invasive agents using ShMOLLI [12] using the Siemens Works-In-Progress MOLLI sequence on identical 1.5 T MR systems (Siemens Avanto, Germany, system software versions VB15 and VB17) using either 16 or 32 channel cardiac coil arrays. Operators were allowed to perform standard cardiac planning with resulting variation in the following image acquisition parameters: Number of Phase Encoding Steps = 105 ± 11 range = 74 to 143 median = 101 ms; TE = 0.5*TR = 1.06 ± 0.01 range = 0.99 to 1.07 median = 1.07 ms; Percent phase field of view = 73 ± 8, range = 51.04 to 98.96, median = 69.79; Acquisition matrix = 192 by 140 ± 15, range = 98 to 190, median = 134; Phase partial Fourier 6/8; Slice thickness = 8 ± 0, range = 5 to 8, median = 8 mm; Minimum TI = 103 ± 5, range = 95 to 125, median = 100 ms, TI increment = 80 ± 0 ms. Imaging was performed with SSFP using flip 35° angle. Each image readout was preceded by 5 ramp up LISA pulses [13], and followed by a single 17.5° pulse at a TR/2 distance. Inversions were performed using a 10ms hyperbolic secant pulse [14]. A complete list of typical acquisition parameters is available in the Online Additional file 1.

### Post-processing and analysis of T1-maps

**Figure 1:**
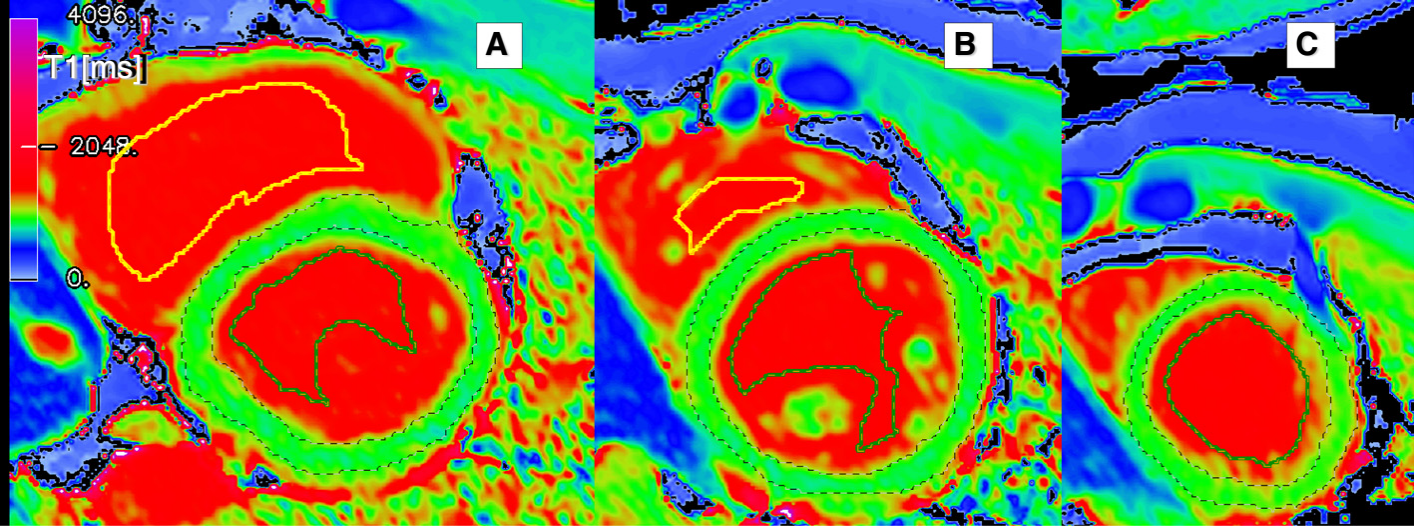
**Typical T1 maps from a single healthy subject. **Basal (**A**), mid-ventricular (**B**) and apical (**C**) short-axis slices. Thin dashed lines denote manually contoured endo- and epi-cardial outlines. Thick coloured outlines mark the left (dark green) and right (yellow) ventricular blood pool, placed within the left- and right-ventricular cavity, respectively, avoiding papillary muscle.

All images were analysed using in-house software written in IDL by SKP (Interactive Data Language, Ver. 6.1, Exelis Visual Information Solutions, Inc., Boulder, USA). Myocardial contours were drawn directly on the T1 maps with full control over image windowing in order to separate consistent myocardial tissue with minimal partial volume of the neighbouring tissues. All contours were reviewed for consistency with further adjustment as deemed necessary by a single observer (SKP). Each MR scan typically provided 3 myocardial slices per subject (average 3.4 ± 1.4, range 1–7 slices) for analysis, representing an average myocardial volume sample of 25 ± 11 ml (~3000 ± 1400 interpolated pixels) per subject. The T1 ± SD per subject for each measurement was calculated as an average using all the slices available for that subject in areas of the left ventricular myocardium, left and right ventricular blood pools (Figure 1).

#### Artefacts and quality assessment

Quality assessment of T1-maps was performed as previously described [15]. Briefly, each T1-map was subdivided into 6 equal segments using the anterior right ventricular-left ventricular insertion point as reference. The presence of off-resonance artefacts and diaphragmatic movement was assessed by examination of the raw T1-weighted SSFP images, while the goodness-of-fit for how well T1 model fitting was achieved for each T1 map was assessed using R^2^ maps. Rejection of segments with suspected artefacts slightly reduced the volume sample to 23 ± 11 ml per individual.

#### Partial volume effect and myocardial midwall T1

The thickness of myocardium was the average distance between epi- and endo-cardial outlines towards the centre of the left ventricle (LV). To study the impact of partial volume on T1 values, the areas marked as myocardium were systematically inflated (by 1–3 pixels, i.e. ~1-3 mm) and eroded (by 1–5 pixels) at both epi- and endo-myocardial outlines and the average T1 ± SD for each measurement re-calculated as above. The tissue sample outlined by myocardial contours following a 1-pixel erosion was defined as the “myocardial midwall”.

### Reproducibility – intra- and inter-centre measurements

Thirty-two additional scans in 21 individuals (total 53 scans) were used to test the reproducibility of T1-mapping. Eighteen subjects were scanned more than once within the Oxford centre over a period of 3 years. For inter-centre comparisons, 9 subjects were scanned in both the Oxford and London centres within 24 hours, while 2 of these subjects were also scanned in both the Oxford and Amsterdam centres within a separate 24-hour period. Overall, the repeated measurements represented ~10% of the datasets from each centre and spanned 435 ± 303 days (median 366, range 1–973). T1 values from these measurements were compared for intra- and inter-centre reproducibility.

### Physiological factors

Primary physiological factors with potential effects on T1 variability were the age and gender of each subject. For the analysis of the primary physiological factors and general population characteristics (Table 1), repeated measurements of an individual were averaged.

**Table 1:** Study population statistics and mean T1 values

	**Females (n)**	**Males (n)**	**P**_**gender**_	**Overall (n)**	**R**_**Age**_
**Myocardial T1 [ms]**	974 ± 23 (173)	950 ± 20 (169)	<0.001	962 ± 25 (342)	-0.21
**Myocardial midwall T1 [ms]**	964 ± 21 (173)	943 ± 19 (169)	<0.001	953 ± 23 (342)	-0.2
**Left ventricle blood [ms]**	1577 ± 70 (173)	1491 ± 55 (169)	<0.001	1535 ± 76 (342)	-0.03
**Right ventricle blood [ms]**	1567 ± 82 (173)	1461 ± 66 (169)	<0.001	1515 ± 91 (342)	-0.04
**Age [years]**	39 ± 14 (173)	37 ± 15 (169)	N/S	38 ± 15 (342)	-
**Myocardial thickness [mm]**	4.8 ± 0.8 (173)	6.0 ± 1.1 (169)	<0.001	5.4 ± 1.1 (342)	0.21
**Heart rate [bpm]**	61 ± 9 (173)	59 ± 9 (169)	N/S	60 ± 9 (342)	0.07
**Hematocrit [%]**	40% ± 3% (32)	43% ± 3% (30)	<0.001	42% ± 4% (62)	0.07
**Height [cm]**	166 ± 7 (167)	178 ± 8 (157)	<0.001	172 ± 10 (324)	-0.08
**Weight [kg]**	66 ± 12 (173)	77 ± 14 (169)	<0.001	72 ± 14 (342)	0.24
**BMI [kg/m2]**	24 ± 4 (167)	24 ± 4 (157)	N/S	24 ± 4 (324)	0.36

Note: P_gender _denotes uncorrected student *t*-test for gender difference. R_Age _– overall correlation with age.

Secondary physiological factors were analysed on a per-measurement basis. These included weight, height, body mass index (BMI), hematocrit, myocardial wall thickness averaged between slices, and average heart rates from sequence timing.

Given that virtually all the studied variables have known primary age and/or gender dependencies, and that male variables generally demonstrated less age-dependency, we chose to equalise the secondary physiological factors to average male values by removing the gender difference and any identified linear trend with age before any subsequent analysis:

$$X_{\text{AGC}}=X+X_{\text{Average}|\text{Male}}-\text{Age}\cdot \text{Slope}\left(X,\text{Age}|\text{gender}\right)-\text{Offset}\left(X,\text{Age}|\text{gender}\right)$$

where *Slope* and *Offset* describe linear regression between variable “X” and age for either gender. “X” may be any T1 or physiological factor. Correction is applied independently of statistical significance of respective regressions. *AGC* stands for “age- and gender-corrected”.

### Statistical analysis

All data are presented as mean ± SD for individuals or measurements as indicated. All differences were assessed using unpaired student t-tests to simulate independent group comparisons. Significance is quoted when probability is less than 0.05 divided by number of simultaneous comparisons in the relevant analysis (Bonferroni correction). Repeated measurements were analysed using the Bland-Altman method [16].

## Results

The overall population statistics are summarised in Table 1. The normal range for myocardial T1 of the entire cohort was 962 ± 25 ms (964 ± 27 ms with inclusion of rejected segments with presumed artefacts; p = n/s) and much shorter than blood T1. LV blood had a longer average T1 than RV blood (1535 ± 76 ms vs. 1515 ± 91 ms, p < 0.002), which was gender-specific to males (p < 0.0001). Overall, myocardial T1 showed a relatively small coefficient of inter-individual variation of 2.2 ± 0.2%, while LV and RV blood T1 showed a larger relative variability of 4.4 ± 0.6% and 5.3 ± 0.7%, respectively. Most of the studied variables showed dependencies on gender and/or age.

### Partial volume effects on T1 estimates

**Figure 2:**
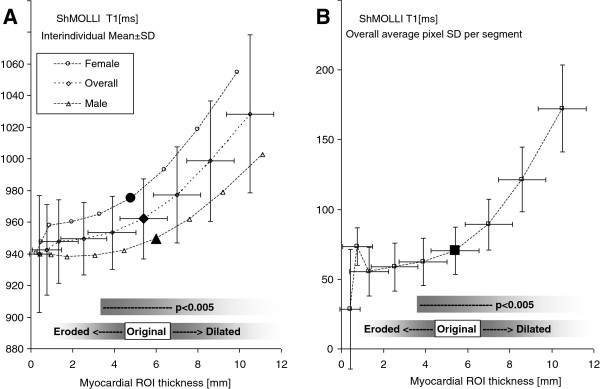
**The effect of partial volume on A) average myocardial T1 and B) average segmental pixel variability. Notes: **Myocardial ROI thickness is calculated as distance between endo- and epi-cardial contours. Filled large symbols represent the T1 derived from myocardial contours drawn manually by the operator (original) compared to systematic erosion or dilatation in 1-pixel increments. P values refer to comparisons between subsequent inflation/erosion steps.

Partial volume effect on myocardial T1 was assessed by changing the average distance between epi- and endo-myocardial outlines from 0.5 to 12 mm (Figure 2) and comparing the average myocardial T1 values from pixels within these contours.

By inflating the myocardial contours, both T1 and its variability increased rapidly due to gross inclusion of neighbouring tissues, particularly blood (with long T1) and smaller areas of pericardial fat, liver and other tissues. While the average T1 value increased by 1.6, 3.8 and 6.9% in successive steps of contour inflation (Figure 2A), there was a much more pronounced increase in T1 variability by 32, 86 and 174% (all p < 0.0005; Figure 2B).

Conversely, erosion of myocardial contours produced an opposite but more limited effect. Only the initial 1-pixel erosion towards the myocardial midwall resulted in a significant 1% reduction in T1 values (p < 0.005). The partial volume effect on T1 reduction was less prominent in men than in women (-0.76% vs. -1.05%) due to the significant difference in the original myocardial wall thickness (Table 1). There was no significant impact with further contour erosion, as shown by diminishing reduction in T1 outside the threshold of significance (with the largest sequential reduction in female T1 of -0.5%, p = 0.25; Figure 2A). There was no added benefit in more aggressive contour erosion beyond 1 pixel from the original contours on decreasing the T1 variability (Figure 2B).

We had therefore applied a 1-pixel erosion to the original, manually-drawn myocardial contours to outline the “midwall myocardial” tissue to minimise partial volume effects whilst still preserving the sampled tissue volume (16 ± 8 ml per individual).

### Reproducibility of T1 measurements – intra- and inter-centre comparisons

**Figure 3:**
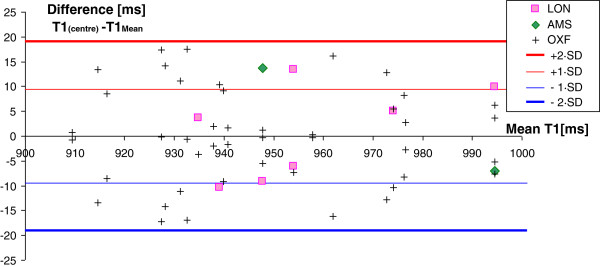
**Intra- and inter-centre reproducibility of myocardial T1 measurements. **Repetition accuracy within the Oxford centre and amongst the three test centres.

Differences in midwall myocardial T1 values on repeated measurements are shown in Figure 3. The average absolute difference between T1 measurements was small (8 ± 5 ms) and the standard deviation of repeats about their averages was ±10 ms. Inclusion of the original edge pixels and segments with artefacts previously rejected increased the T1 variability by only ~1 ms, each. There were no statistically significant differences between errors within and between centres and there was no significant dependence between T1 differences and the time between measurements. LV blood T1 repeats showed an average absolute difference of 19 ± 14 and SD ± 23 ms, which was slightly less than for RV blood T1 (23 ± 16, SD ± 28 ms).

### Primary physiologic factors: age and gender influence on myocardial and blood T1 values

**Figure 4:**
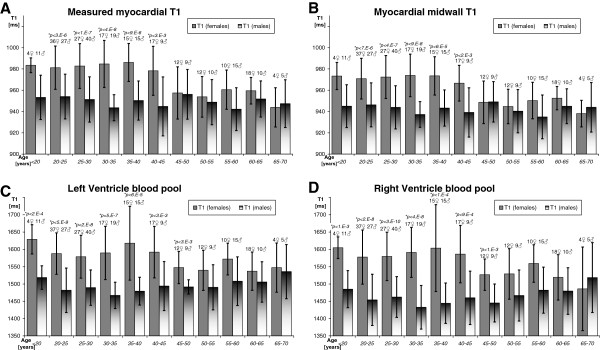
**Age- and gender-dependence of myocardial and blood T1. A**) Measured myocardial T1 within manually drawn myocardial contours demonstrated a small elevation of T1 in young females. **B**) Myocardial midwall T1 (see partial volume section), indicated a similar persistent gender difference, albeit at a slightly lower T1 likely due to reduction of blood partial volume. **C**) Left Ventricle blood T1. **D**) Right Ventricle blood T1. **Note: **Unpaired student T-test p-values are marked above each bar for age-groups when Bonferroni-corrected significance threshold is achieved for gender difference.

The average myocardial and blood T1 values are presented in Figure 4 according to age groups.

In males, there was no age-dependency of either myocardial or blood T1 values on either individual or age-group regression analysis (p > 0.3).

In females, on the contrary, myocardial T1 was consistently higher than males up to the age of 45 years, after which there was convergence with no significant difference from male T1. This resulted in a significant overall trend towards female myocardial T1 decreasing with age (p < 0.01), with the slope ranging from -0.7 ms/year (individual midwall myocardial T1) to -0.8 ms/year (group average of midwall myocardial T1). Blood T1 in the LV and RV showed similar age- and gender-dependency (Figure 4 CD). While there were no significant trends in males, LV blood T1 in females decreased by -1.1 to -1.2 ms/year based on the individual and the age-group average estimates (p < 0.01). RV blood T1 also decreased by -1.6 to -2 ms/year (on both individual and age-group regression analysis p < 0.01).

### Secondary physiologic factors: influence on myocardial and blood T1 values

**Figure 5:**
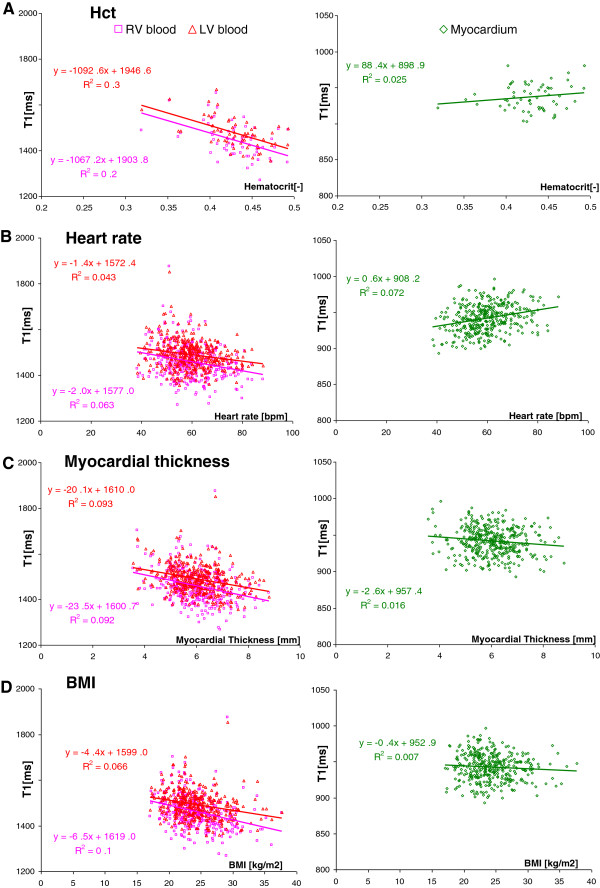
**The effects of common physiological parameters on the myocardial midwall T1 and blood T1 after correcting for age and gender differences. **(**A**) Blood hematocrit is the principle driving force of blood T1s, but not myocardial T1, variation. (**B**) Increase in heart rate is associated with an increase in myocardial T1 and a decrease in blood T1s. (**C**) There is no relation between myocardial thickness and myocardial T1, but blood T1s change in opposite direction. (**D**) Increased body size does not influence myocardial T1, but decreases blood T1s. **Note: **statistical significance of marked correlations reaches Bonferroni-corrected significance of p < 0.002 (p < 0.05/18 comparisons including height and weight not shown here) for r^2^ > 0.15 (n = 62, **A**) and r^2^ > 0.026 (n = 374, **B-D**).

As intended, age- and gender-correction (AGC) removed any inherent relationships of a variable to age and gender, and most interdependencies amongst the physiological factors studied. Specifically, after AGC, heart rate did not correlate with any other physiological factor; only myocardial thickness correlated with height and BMI, but not weight. Here, we present the effects of secondary physiologic factors on myocardial and blood T1, both before and after AGC, the latter of which is summarised in Figure 5 and Table 2.

**Table 2:** Summary of physiological effects on ShMOLLI T1 measurements

**Physiologic Factor**	**Myocardial T1**	**Blood T1**
Gender (female, <45 years)	+24 ms	+130 ms
↑ Age (females only)	↓ 8 ms/10 years	↓ 20 ms/10 years
↓ Hematocrit	-	↑↑ 11 ms/%
↑ Heart rate	↑ 6 ms/10 bpm	↓ 20 ms/10 bpm
↑ Myocardial thickness	-	↓ 23 ms/mm
↑ Height	-	-
↑ Weight	-	↓ 20 ms/10 kg
↑ BMI	-	↓ 7 ms/kg/m^2^

#### Hematocrit

T1 is related to the water content in any tissue [1] and specifically in blood [17], which was strongly supported by a high negative correlation between blood hematocrit (Hct) and blood T1 (r = -0.7), both before and after AGC (r < -0.45, p < 0.0003, Figure 5A). There was no significant link between myocardial T1 and Hct either before or after AGC (Figure 5A).

#### Heart rate

Before AGC, myocardial T1 increased with increasing heart rate (for males, females and overall, all r > 0.2, p < 0.004). After AGC, this relationship strengthened to r = 0.27 with a slope of 0.56 ms/bpm. On the contrary, blood T1 decreased with increasing heart rate (Figure 5B).

#### Myocardial wall thickness

Before AGC, there was a moderate negative correlation between myocardial thickness and midwall myocardial T1 derived from 1-pixel erosion of manually-placed contours (r = -0.33, slope = -8 ms/mm), but this did not achieve Bonferroni-corrected significance after AGC (Figure 5C). Interestingly, AGC dependence of this relationship reached significance (r = -0.17) for myocardial T1 derived from the original contours before erosion, which further strengthened with inflating the contours (r = -0.26-0.29) but weakened (r = -0.1) with further erosion (2 pixels), indicating that the edge partial volume effect was the primary source of this dependency.

#### Body size

Before AGC, there were significant negative correlations between midwall myocardial T1 and height (r = -0.28), weight (r = -0.32) and BMI (r = -0.17, all p < 0.001), but all were either rejected (height) or reduced to trend level (weight and BMI) after AGC (Figure 5D).

For blood T1, there were significant negative correlations between body height, weight and BMI for LV blood T1 (r < -0.25) and RV blood T1 (r < -0.31), both before and after AGC (Figure 5D).

#### T1 inter-dependence between blood and myocardium

Blood T1 of LV and RV are strongly correlated with each other independent of AGC (r > 0.9, p < 0.0001). There were significant correlations between blood and myocardial T1 in males, females, overall, before and after AGC. After AGC, the slopes of the relationship between myocardial and blood T1 (0.11 for T1_LV,AGC_ and 0.09 for T1_RV,AGC_) is consistent with the resting partition of blood volume in myocardial tissue (all r > 0.3, p < 0.0001) [18].

## Discussion

In this work, we have presented the largest database to-date on normal myocardial T1 relaxation times measured using the ShMOLLI method in three clinical centres. We demonstrated that native myocardial T1 is a robust and reproducible biological parameter with a narrow normal range. The primary technical factor that may influence myocardial T1 analysis is the partial volume effect when there is gross over-inclusion of neighbouring tissue at the image post-processing stage. Hierarchical analysis of physiologic influences showed that females have higher myocardial T1 values compared to males up to the age of 45 years, after which there is no difference from male T1. Following compensation for age and gender, only heart-rate remained as a secondary physiologic factor with a small effect on myocardial T1 values.

### Myocardial T1-mapping as a robust biomarker

The results of this study affirmed the reproducibility and robustness of T1-mapping by comparing data amongst three clinical MR centres. The stability of the method is based on data that withstood the tests of time, various software upgrades, MR scanners in different centres, multiple operators, choice of available 16 and 32 channel coils, positioning, shimming strategies and other study-dependent factors that are beyond practical control. Despite this, the consistency observed was not far from the initial intra-scan repeatability of our method of about 1.6% at 1.5 T [12].

Given that T1-mapping serves as an objective method for myocardial characterisation that is less prone to observer bias, we examined how operator placement of myocardial contours and partial volume may affect T1 values. Our analysis suggested that the human operator places endo- and epi-cardial contours just on the edge of the myocardial interface with neighbouring tissue, with a relatively small 1% overestimation from the midwall myocardial T1. In other words, tightening or loosening of endo-and epi-cardial boundaries around human-drawn contours produces only a ±2% variation in myocardial T1. Thus, intra-centre post-processing of T1-maps with consistent analysis protocols using midwall myocardial T1 values should not suffer from any major impact from partial volume effects. However, for inter-centre or inter-study comparisons, it may be helpful to quote myocardial thickness when reporting myocardial T1 values. This is due to the fact that 1-pixel erosion indicated in this study may not be appropriate to myocardial contours drawn according to different guidelines, in which case the reference point in Figure 2, and the amount of erosion needed to obtain midwall myocardial sample will change. Specifically, care has to be applied when comparing the results of studies with wide myocardial contours visibly inside ventricular blood pool [19] with those characterising a narrow midwall sample [20,21]. The latter approach is more accurate and appropriate for research studies to examine underlying disease processes and is needed especially in models of diffuse myocardial disease to improve measurement consistency. On the other hand, maximising myocardial coverage may be desirable for distinguishing subendocardial or subepicardial disease for clinical diagnosis. Under such circumstances the experienced operator should be able to distinguish pathology from gross over-inclusion of neighbouring tissues. All those aspects are helped by the standard available on the scanner two-fold image interpolation, which we use consistently across all our studies prior to T1 fitting in order to improve the final T1 image quality. Potential further developments, such as image co-registration, may alleviate partial volume effects due to within-slice motion [22]. Effects related to through-slice motion and partial volumes are more complicated and need further study.

The primary physiologic determinants of myocardial T1 examined were age and gender, with the largest effect seen in younger females, showing an average T1 prolonged by 24 ms compared to males. While changes in myocardial and blood T1 in women appeared to show a sharp transition at around 45 years of age, a possible link to menopause needs to be confirmed. Given that the residuals of simple regression were within the range of variability according to age groups, modelling the age-dependence of T1 with higher order non-linear models is unwarranted without a clear hypothesis to test.

Since the largest age-related effect corresponded to a T1 change of only 7–8 ms/decade of age (i.e., ±2% variation between 20–70 years-old), ShMOLLI T1 mapping may be used without age- and gender-correction for the detection of major myocardial events such as acute infarction, edema or myocarditis in which myocardial T1 increases substantially [3,5,15,23] For instance, in acute myocardial infarction, native myocardial T1 of acutely ischemic segments showed an average T1 increase of 21% (1197 ± 76 ms vs. 987 ± 34 ms in remote areas) [4] and as high as 39% (1624 ms vs. 1166 ± 60 ms in controls at 3 T) [3]. Similarly, in acute myocardial oedema at 1.5 T, myocardial T1 of affected segments showed an average value of 1113 ± 94 ms and as high as 1375 ms compared to 944 ± 17 ms in controls, representing an 18% and 45% increase, respectively [15]. However, for diffuse or low grade forms of cardiovascular pathology or when volumetric quantification of affected myocardium is desired, it may be appropriate to use age- and gender-matched controls to maximise sensitivity to detect subtle disease. In the future it would be feasible to perform age- and gender-compensation directly on the scanner console based on input of subject characteristics. More accurate models may be constructed with further research, incorporating physiologic parameters such as hematocrit, weight and height and other variables.

Overall, we found that blood T1 was more sensitive than myocardial T1 to the secondary physiological factors. While there are biologically plausible reasons for the observed dependencies of blood T1 on the hematocrit (water content), weight and BMI (lipids in blood), we do not see a clear reason for heart-rate dependency. It remains to be established whether this dependency is related to any aspect of blood physiology or arises from inflow or other technical issues not yet identified from phantom studies. The relationship between myocardial T1 and heart-rate is relatively flat at 5.6 ms/10 bpm (±1.5% variation within the range of 50–100 bpm).

### Implications and future directions for clinical application of T1-mapping

The results of this study showed that the potential influence of any single factor tested was within 2% of the average myocardial T1 values. While errors can be additive, it is foreseeable that primary physiological factors such as age and gender can be matched. Similarly, differences in groups can be compensated for using the correction factors summarised in Table 2. Most corrections apply to blood T1, whereas the myocardial T1 has much shorter list and relatively weaker corrections. Such a tight range of myocardial T1 variability establishes T1-mapping as one of the most robust biomarkers, especially within MR imaging methodology.

### Accuracy of T1 measurements

From the technical standpoint, all numeric results presented in this study are estimates subject to measurement bias and variability related to the choice of method. In particular, T1-mapping using ShMOLLI was introduced as a clinically-applicable method that improved on the longer breath-hold duration and known heart rate sensitivity of conventional MOLLI and its variants [12,24,25]. Our T1 values results are corrected for Look Locker effects in line with previous work [12,24] but we did not correct for the underestimation of ShMOLLI T1 by 4% compared to the reference T1 using inversion recovery, even though it was verified in both in simulation and the phantom experiments [12]. This serves to preserve the similarity of myocardial ShMOLLI T1 estimates to the legacy MOLLI T1 at 1.5 T and 3 T under reasonably normal heart rate conditions [12].

Such comparisons do not include high T1 values of blood and in significant tachycardia where MOLLI variants exhibit an increasing heart-rate dependence [12,25]. Also, we specifically exclude any comparisons to the assorted corrections of MOLLI heart rate dependencies that recently appeared in literature, but for which no consensus has yet formed. In particular the early measures of T1 dependence on heart rate in small (N = 15) group of normal control volunteers indicated that MOLLI T1 *increases* with a slope of 2.7 ms/BPM [26]. Counteracting this by correction [27] is thus expected only to deepen the confirmed underestimation errors [12,25]. Recently Kawel et al. in the appendix to their JCMR publication [21] described a complicated correction method resulting in an increase in T1 estimates by about 10% over prior MOLLI and ShMOLLI T1 results in 3T [12]. It is unclear if exactly these precise formulas were also used by Lee et al., who also quotes high normal myocardial T1 of 1315 ± 39 ms at 3 T [25]. However, when corrections are removed the resulting T1 falls back into the range reported previously by us using MOLLI and ShMOLLI in 3T [12]. Surprisingly, Kawel et al. indicates that these corrections are only applicable to 3T but without providing reasoning or absolute T1 estimates [28], even though elsewhere they report relatively high T1 of ~1010 ± 36 ms also at 1.5 T without mentioning any respective corrections [20].

The lack of full detail, applicability criteria and validation of such attempts to improve accuracy makes myocardial T1 data hard to compare across the literature. Furthermore, the recent experiments on the residual sensitivity to tissue T2 [29] and the effects of imperfect adiabatic inversion pulses [30] offer yet further insights into the complicated issues regarding the absolute accuracy of T1 measurements. Clearly, the technical aspects of T1 measurement accuracy require much further arduous work on the impact of manifold choices across cardiac T1-mapping and even at the root of the reference validation methods. Perfect measurement of T1 is highly desirable for physicists and future sequences may provide improvements, but currently there is no indisputably accurate in-vivo myocardial T1 measurement method. However, there is a growing range of clinical applications for non-invasive T1-mapping as a biomarker with stable and reliable normal reference range, such as addressed in this paper. Therefore we specify the T1-mapping method used to measure the T1 values across this paper as “ShMOLLI T1” or “T1 using ShMOLLI” referring to documented methods [12] to avoid confounding the literature with the apparent large variation in normal “MOLLI-related” T1 estimates.

### Other limitations

The multicentre data in this study was reviewed and analysis performed by a single observer (SKP). While re-analysis of the whole material by another observer would be impractical given its quantity, our prior analysis showed intra- and interobserver differences to be small at ±0.5% [15], which is significantly less than other major sources of variation.

MOLLI methods are SSFP-based and thus are sensitive to certain imaging artefacts, which were primarily assessed by visual inspection and rejection. In this study, we aimed to establish a normal range for native myocardial T1 without artefact influence and thus removed about 10% of the myocardial tissue sample volume; however, it was reassuring that re-inclusion of rejected data did not significantly influence the population myocardial T1 average. Thus artefacts are unlikely to affect the clinical applicability of the method when the trained operator is able to identify problematic measurements and to repeat scans with frequency offset or improved shimming, as is frequently done for other common CMR methods.

In this study we are aware of small dependencies that arise from predictable changes in imaging parameters, such as the length of the acquisition, number of k-space lines and MOLLI timings that are automatically adjusted with image planning. These contribute to the presented relationships, but given the complicated dependencies between numerous MR physics factors, the dedicated analysis of underlying mechanisms has been delegated to future research. From the insight offered by the variation in the imaging parameters presented in the MR acquisition section, we estimate that technical factors do not substantially affect the presented relations as to have a significant effect on ShMOLLI T1 performance in the detection of large acute T1 changes. In the future, the application of additional corrections, or keeping all image acquisition parameters as constant as possible, may increase sensitivity to small differences between clinical groups.

## Conclusion

Native T1-mapping using ShMOLLI at 1.5 T appears to be a highly reproducible, robust and stable biomarker for characterising the human myocardium. Native myocardial T1 exhibit a narrow normal range with limited variability related to common technical and physiologic factors, rendering it a potential method for quantitative disease detection without the need for exogenous contrast agents. The main technical factor affecting myocardial T1 analysis was partial volume effect which is more pronounced in thinner myocardium, but can be alleviated by careful placement and/or erosion of myocardial contours. The primary physiological factors affecting native myocardial T1 are female gender, female age and heart rate. The study of cardiac conditions with more subtle changes, such as diffuse fibrosis or small focal changes, may benefit from age and gender matching between patients and controls. As the accuracy of current T1 measurement methods remains to be established, this study does not claim to report an accurate measure of T1, but that ShMOLLI is a stable and reproducible method for T1-mapping.

## Competing interests

US patent pending 61/387,591: SKP, MDR and AG. *SYSTEMS AND METHODS FOR SHORTENED LOOK LOCKER INVERSION RECOVERY (Sh-MOLLI) CARDIAC GATED MAPPING OF T1*. September 29, 2010. All rights transferred to Siemens Medical.

## Authors’ contributions

SKP provided the concepts, performed data analysis and drafted the manuscript. VMF contributed data, processed images and contributed significantly to the drafting of the manuscript. AJL, NABN, contributed data, processed images. RB, CH, MBMH, DMS, VM, SW, ML contributed data. MBMH, TK, JCM, SN, PL, MDR supervised projects contributing data and acted in last author capacity. All authors read, commented or edited the manuscript, and approved the final version.

## Supplementary Material

Additional file 1 Typical ShMOLLI parameter list. Click here for file
