# Supplementary material for: Normal variation of magnetic resonance T1 relaxation times in the human population at 1.5 T using ShMOLLI
Source: J Cardiovasc Magn Reson. 2013 Jan 20;15(1):13. doi: 10.1186/1532-429X-15-13 (PMC3610210; doi:10.1186/1532-429X-15-13)
Supplement: Additional file 1 — Typical ShMOLLI parameter list. [file 1532-429X-15-13-S1.pdf]

\\USER\People\StefanP\ShMolliTemplates1.5T\ShortMolli_Oxford

TA: 6.3 s PAT: 2 Voxel size: 1.8×1.8×8.0 mm Rel. SNR: 1.00 USER: CV_molli_561

Properties

Prio Recon	Off
Before measurement	
After measurement	
Load to viewer	On
Inline movie	Off
Auto store images	On
Load to stamp segments	Off
Load images to graphic segments	Off
Auto open inline display	Off
Start measurement without further preparation	On
Wait for user to start	On
Start measurements	single

Routine

Slice group 1	
Slices	1
Dist. factor	25 %
Position	L90.7 A37.3 H25.7
Orientation	S > C43.0 > T25.7
Phase enc. dir.	A >> P
Rotation	0.00 deg
Auto	Off
Phase oversampling	0 %
FoV read	340 mm
FoV phase	68.8 %
Slice thickness	8.0 mm
TR	198.77 ms
TE	1.07 ms
Averages	1
Concatenations	1
Filter	Raw filter, Distortion Corr.(2D)
Coil elements	BA1-4;BP1-4

Contrast

Magn. preparation	Non-sel. IR
TI	100 ms
Flip angle	35 deg
Fat suppr.	None
Restore magn.	On
Averaging mode	Short term
Reconstruction	Magn./Phase
Measurements	1
Multiple series	Off

Resolution

Base resolution	192
Phase resolution	100 %
Phase partial Fourier	6/8
Trajectory	Cartesian
Interpolation	On
PAT mode	GRAPPA
Accel. factor PE	2
Ref. lines PE	24
Matrix Coil Mode	Auto (Triple)
Reference scan mode	Integrated
Image Filter	Off
Distortion Corr.	On
Mode	2D
Unfiltered images	Off
Prescan Normalize	Off
Normalize	Off
B1 filter	Off
Raw filter	On
Intensity	Weak
Slope	25
Elliptical filter	Off
POCS	Off

Geometry

Multi-slice mode	Sequential
Series	Interleaved
Special sat.	None

System

Body	Off
BA1	On
BA2	On
BA3	On
BA4	On
BP1	On
BP2	On
BP3	On
BP4	On
Positioning mode	REF
Table position	F
Table position	26 mm
MSMA	S - C - T
Sagittal	R >> L
Coronal	A >> P
Transversal	F >> H
Save uncombined	Off
Coil Combine Mode	Adaptive Combine
AutoAlign	---
Auto Coil Select	Default
Shim mode	Tune up
Adjust with body coil	Off
Confirm freq. adjustment	Off
Assume Silicone	Off
? Ref. amplitude 1H	0.000 V
Adjustment Tolerance	Auto
Adjust volume	
Position	Isocenter
Orientation	Transversal
Rotation	0.00 deg
R >> L	350 mm
A >> P	263 mm
F >> H	350 mm

Physio

1st Signal/Mode	ECG/Trigger
Average cycle	340 ± 10 ms
Captured cycle	-not set-
Acquisition window	829 ms
Trigger pulse	1
Trigger delay	340 ms
Segments	78
Phases	1
Tagging	None
Dark blood	Off
Cine	Off
Inline ventricular function	Off

Inline

Subtract	Off
Std-Dev-Sag	Off
Std-Dev-Cor	Off
Std-Dev-Tra	Off
Std-Dev-Time	Off
MIP-Sag	Off
MIP-Cor	Off
MIP-Tra	Off
MIP-Time	Off
Save original images	On

Sequence

Introduction	Off
Dimension	2D
Reordering	Linear
Asymmetric echo	Allowed
Bandwidth	898 Hz/Px
Optimization	Min. TE TR
Echo spacing	2.6 ms
Sequence type	Trufi
Define	Shots
Shots per slice	1
Trufi delta freq.	0 Hz
RF pulse type	Fast
Gradient mode	Fast
Excitation	Slice-sel.
Flip angle mode	Constant
Mode	MOLLI
Inversions	3
Acquisition 1st Inv	5 HBs
Acquisition 2nd Inv	1 HBs
Acquisition 3rd Inv	1 HBs
Acquisition 4th Inv	0 HBs
Acquisition 5th Inv	0 HBs
Recovery 1st Inv	1 HBs
Recovery 2nd Inv	1 HBs
Recovery 3rd Inv	0 HBs
Recovery 4th Inv	0 HBs
Recovery 5th Inv	0 HBs
TI	100.0 ms
TI increment	80 ms
MOLLI Trigger delay	500 ms
